# Analysis of peripheral artery velocity tracing in a porcine model

**DOI:** 10.2478/v10019-011-0004-9

**Published:** 2011-03-15

**Authors:** Qingxin Meng, Weiwei Ding, Bin Yang, Ninghua Fu, Guangming Lu

**Affiliations:** 1 Department of Ultrasound, Jinling Hospital, School of Medicine, Nanjing University, Nanjing, China; 2 Research Institute of General Surgery, Jinling Hospital, School of Medicine, Nanjing University, Nanjing, China; 3 Department of Medical Imaging, Jinling Hospital, School of Medicine, Nanjing University, Nanjing, China

**Keywords:** velocity tracing, resistance, effective circulating blood volume, elastic recoil, principal wave, backward wave

## Abstract

**Background:**

The aim of the study was to trace the peripheral artery velocity with ultrasound in pigs and provide inference on diagnosis of the type, location and severity of vascular diseases.

**Materials and methods:**

Limb tightening, adrenaline administration and arterial wall pinching were performed independently in six pigs, and then the evolution of the external iliac artery or femoral artery velocity tracing were monitored.

**Results:**

With the increase of the extents of hindlimb tightening, peak systolic velocity (PSV) of ipsilateral external iliac artery turned from 36.33±1.77 cm/s to 59.72±2.67 cm/s, minimum post-principal wave velocity (MPV from 13.68±1.11 cm/s to −7.48±0.82 cm/s, peak diastolic velocity (PDV) from 19.31±0.86 cm/s to 8.98±0.45 cm/s, and, end diastolic velocity (EDV) from 13.2±0.45 cm/s to 0. With the increase of the dose of the epinephrine injection, PSV increased from 36.33±1.77 cm/s to 43.97±2.15 cm/s but then decreased to 35.43±3.01 cm/s, and MPV negatively increased to −23.53±0.82 cm/s after decreasing from 13.68±1.11 cm/s to 0. PDV and EDV gradually decreased to zero. With the increase of the stenosis severity in the abdominal aortic wall pinching, PSV was reduced and had a linearly negative correlation with the stenosis severity (R=0.983, R2=0.967). MPV gradually increased, and its direction reversed when the stenosis severity increased, then diminished when the blood flow was occluded by more than 2/3.

**Conclusions:**

The formation of peripheral artery velocity is the result of concurrent effects of cardiac ejection, vascular resistance, effective circulating blood volume and elastic recoil. Vascular resistance exerts pronounced effects on the diastolic waveform, and the occurrence of backward wave indicates that the downstream circulation resistance significantly increases.

## Introduction

The morbidity and mortality of cardiovascular diseases are growing with the increase of hypertension, hyperglycaemia and hyperlipidemia. They are still the main causes of death and the determinants of the decreased quality of life. Most of these diseases can cause the alteration of local or distant hemodynamics because of lumen stenosis or dilatation.^1^ The changes in the hemodynamics may alter the magnitude and distribution of regional shearing force, damage the vascular endothelium cells and result in local intimal proliferation, finally leading to the formation of atheromatous plaques. The shape, location and stability of atheromatous plaques are closely associated with the way in which regional shearing force acts, playing an important role in thrombosis.^2^–^7^ It is important, as early as possible, to identify lumen stenosis or dilatation, analyze the changes in hemodynamics, and applied effective strategies to control its aggravation or even eliminate the lesions.^8^,^9^

Some researchers have studied the changes in hemodynamics caused by arterial stenosis, and proposed the ways in which the hemodynamics is evaluated in experiments *in vivo* or *in vitro.*^4^,^10^–^13^ Ultrasound can be used to detect blood vessels in every direction and is dynamical, non-invasive, economical and convenient modality.^9^,^14^ Therefore, hemodynamics can be monitored with ultrasound through the velocity tracing. Apart from pulsatility index (PI) and resistant index (RI)^15^–^18^, peak systolic velocity (PSV) and ratio of PSV at stenosis to PSV at proximal artery are generally regarded as the important indexes in predicting stenosis and its severity.^19^–^24^ However, the valve of velocity is related to many factors such as the site of sampling and the correction angle.

In addition, content in the enteric cavity, scar, muscles, fat tissues and tissue oedema may lead to pronounced acoustic attenuation in deep tissues resulting in ambiguous image of blood vessels. It is critical to determine the site of arterial lesion and its severity in the upstream or downstream of the detection site according to the alteration of local velocity, which is superficial or limpid to be revealed. The waveform of “*tardus-parvus*” has been used to detect serious stenosis in the upstream of renal, hepatic and intracranial artery^29^,^30^, and similarly, the velocity of extremity arteries may also be altered strikingly if there is serious stenosis in the upstream.^28^–^30^ Not only the morphous and the level of stenosis, but the pressure, velocity, physical property and resistance of vessels are crucial factors involving in the changes in hemodynamics in the upstream or/and downstream of stenosis.^11^,^34^–^40^

To investigate the mechanisms and contributing factors underlying the formation of peripheral artery velocity, we analyzed the evolution of external iliac artery and femoral artery velocity through tightening limb, administration of adrenaline or pinching the lateral walls of the end of abdominal aorta in pigs. Thus, we determined the type, location and severity of vascular diseases according to local hemodynamics detected by velocity tracing.

## Materials and methods

### Animals

Six female or male juvenile susscrota domesticas weighing 24 ± 1 kg (range: 20∼26 kg) were purchased from the Animal Experiment Center of Jinling Hospital. The whole study was approved by the Animal Research Committee of Jinling Hospital. All procedures were carried out in accordance with the “Principles of Laboratory Animal Care” (NIH publication No.85-23, revised 1985).

### Main instruments

The haemostatic clip with concave and convex *dentes* (lot number: X20870) was purchased from Surgical Instruments Factory, Shanghai Medical Instruments (Group) Corp. Ltd., the micro-pump from B. Braun AG, and the animal monitor from Spacelabs Healthcare, Inc. The ultrasonic instrument was purchased from GE logiq I, with the frequency of 10 MHz.

### Animal processing

The animals were fasted with access to water *ad libitum* for 24 h before the operation, and housed in a room with controlled temperature (28°C). After an overnight fast, the swine was injected with ketamine (20 mg/kg). Droperidol and atropine (0.06 mg/kg), and then fixed in a supine position. After endotracheal intubation and ventilating mechanically, anaesthesia was remained with intravenous injection of 150 μg/kg/min propofol (Disoprivan 2%, emulsion, Astra Zeneca, Wedel, Germany) and bolus injection of 2∼5 μg/kg fentanyl (Janssen Cilag, Neuss, Germany). The vital signs were closely monitored, and the anaesthesia was adjusted according to the blood pressure aiming to ensure the stable circulation.

The hindlimbs were tightened with gauze until blood flow signal was undetectable by ultrasound in the downstream of pressure point, which mimics femoral artery stenosis to different extents. Then, the velocity in the upstream (external iliac artery) was monitored.

Epinephrine administration; about 1/3, 1/2 and 1 dose of ampoule epinephrine hydrochloride (1 ml: 1 mg) were administered intravenously in sequence. The external iliac artery velocity was determined after every injection until it returned to that before experiment. Subsequently, the dose was adjusted and the experiment continued.

### Abdominal aortic wall pinching

After anaesthesia, the animal was fixed in a supine position. After sterilizing and skin preparation, laparotomy was performed and the end of abdominal aorta was exposed. The blood vessels were covered with warm and moist saline gauze after the separation. The end of abdominal aorta served as stenotic site. The blood flow of abdominal aorta was blocked with a haemostatic clip. Then, abdominal aortic wall was partially pinched. Finally, the haemostatic clip was released slowly. This process can produce 1/3, 1/2, 2/3 and 3/4 occlusion in sequence which was demonstrated by the detection of internal diameter of lumen by ultrasound). All haemostatic clips were released each time and the following experiment was carried out after the velocity of common femoral artery returned to that before operation.

Animals were sacrificed by intravenous injection of 100 mg/kg pentobarbital sodium at the end of experiments.

### Instrument regulation and observation indexes

Two-dimensional images could clearly display the vascular lumens. In the colour Doppler flow imaging, the blood flow signals exactly suffused the vascular lumen with soft colour. The sampling frame steer was consistent with the direction of blood stream, the sampling gate located at the center of lumen whose width was 2 mm, and the correction line paralleled with the direction of bloodstream.

A group of similar velocities in different cardiac cycle were detected. Three velocities were randomly selected and calculated. The average represented the velocity of external iliac artery or femoral artery in the state. The PSV, minimum post-principal wave velocity (MPV, peak reverse velocity was considered as MPV in two-phase velocity tracing), PSV/MPV (P/M), MPV/PSV (M/P), peak diastolic velocity (PDV), and end diastolic velocity (EDV) were used to estimate the hemodynamics of external iliac artery or femoral artery.

### Statistical analysis

All quantitative data were presented as means±standard deviation (±s) and processed with SPSS 13.0 statistic software package. Comparisons between multiple groups were done with one way analysis of variance. Intra-group comparisons were performed with LSD test at the homogeneity of variance (the size of test was 0.10), or with Tamhane’s-t test at the heterogeneity of variance. A value of *P* < 0.05 was considered statistically significant.

## Results

### Alteration of velocity wave

**FIGURE 1.**
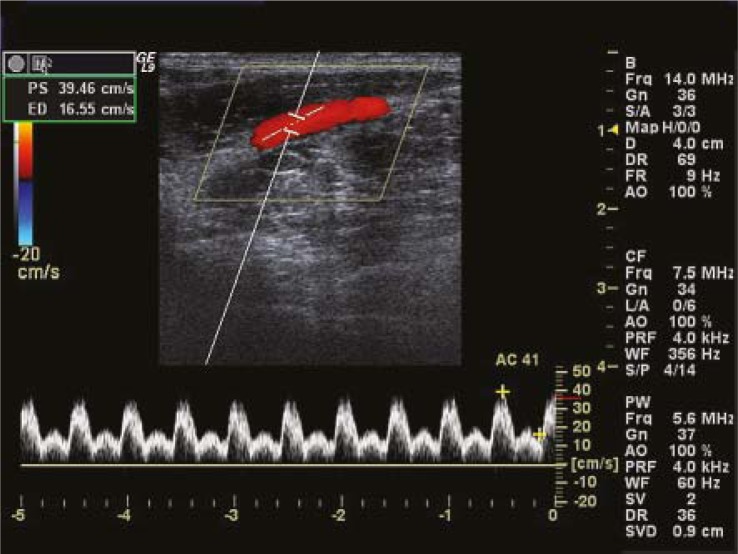
Velocity wave of external iliac artery after anaesthesia.

**FIGURE 2.**
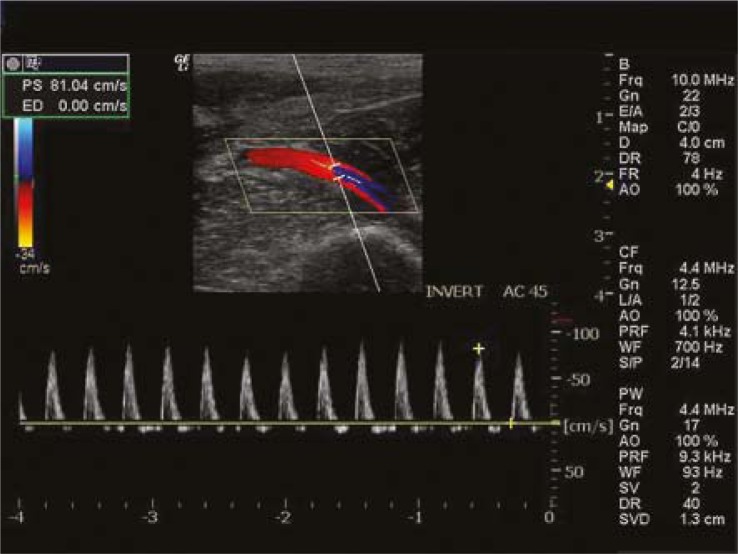
Velocity wave of common femoral artery after anaesthesia.

After successful anaesthesia, the waveform of external iliac artery velocity was composed of systolic principal wave with steep ascent and descent, and diastolic wave with persistent low-amplitude fluctuation (Figure 1). The waveform of common femoral artery velocity consisted of systolic principal wave with steep ascent and slow descent, and diastolic wave with persistent low-amplitude fluctuation backward (Figure 2).

**FIGURE 3.**
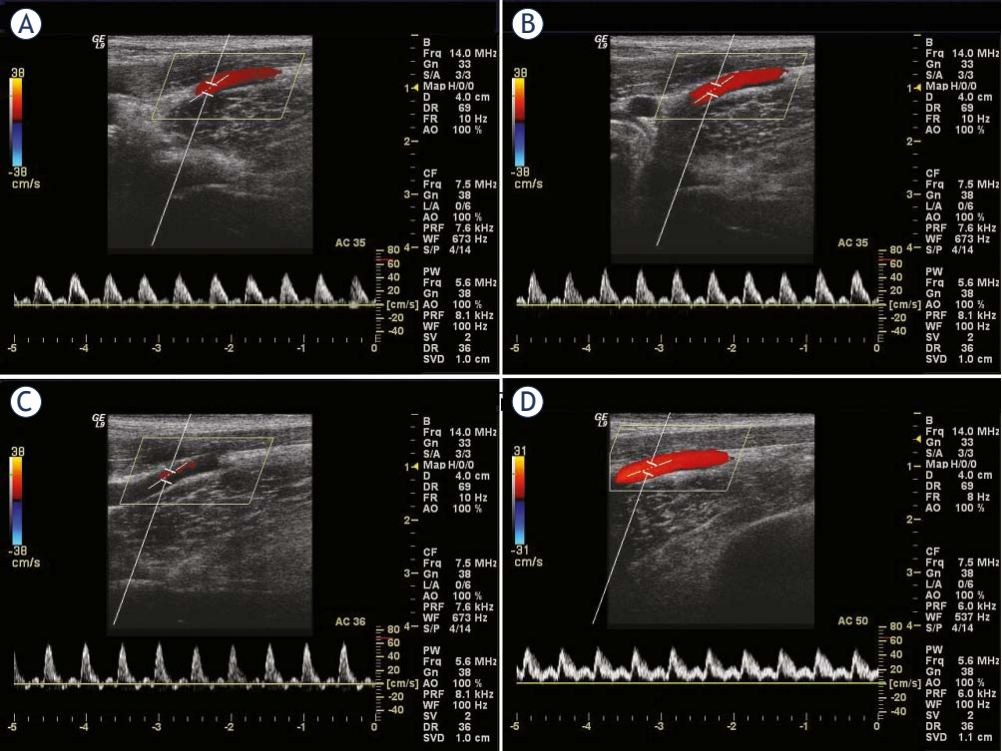
Velocity wave (A–C) of external iliac artery with the increase of pressure in limb tightening; D: velocity wave of external iliac artery after removal of pressure.

In terms of the velocity of external iliac artery during the limb tightening, with the increase of pressure, the systolic waveform tended to be acuminate, and the amplitude of diastolic wave was lowered. MPV gradually diminished to zero, and subsequently, the backward wave emerged with the increase of wave amplitude. EDV dropped to zero by degrees. Under the pressure to certain extent, the triphasic wave appeared and resembled the wave of artery of lower extremity in health adults. The waveform gradually returned to that before experiment after the pressure was removed (Figure 3).

**FIGURE 4.**
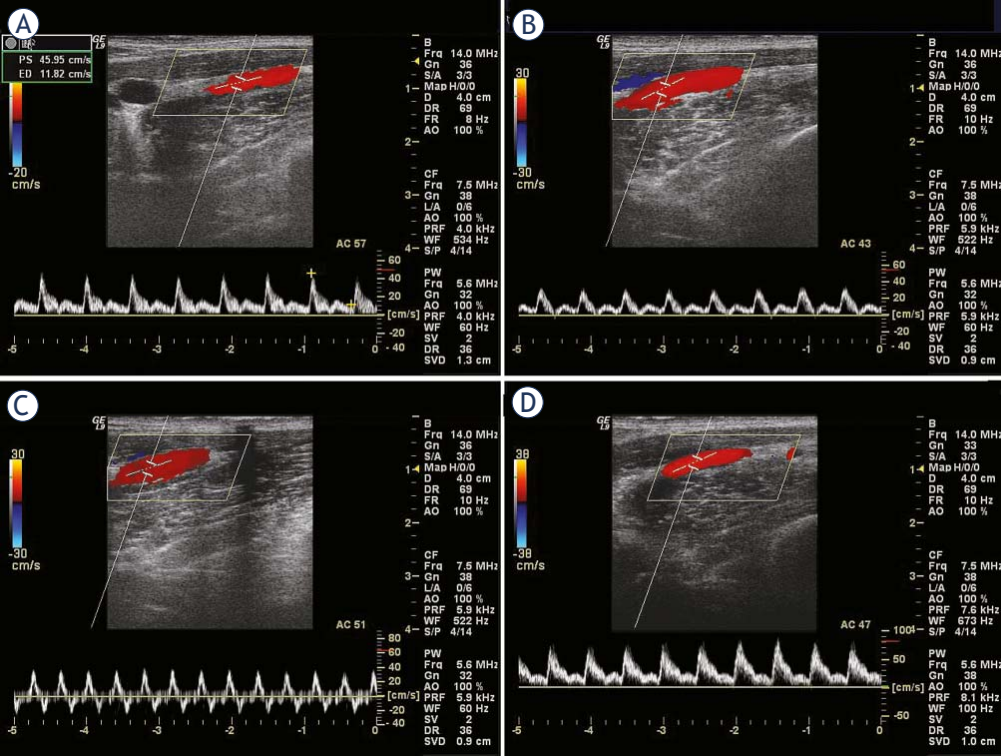
Velocity wave (A–C) of external iliac artery after epinephrine administration. A: 1/3 ampoule, B: 1/2 ampoule, C: 1 ampoule, D: soon after injection discontinuation.

For the velocity of external iliac artery after the intravenous injection of epinephrine, with the increase in dose, the changes in waveform were similar to those after limb tightening, but these changes were more obvious. After the injection of 1 ampoule epinephrine hydrochloride, the waveform presented diphasic and consisted of systolic positive principal wave and diastolic negative wave. However, after the injection discontinuation, the waves were at opposite direction at several time points when compared with waves before the experiment (Figure 4).

**FIGURE 5.**
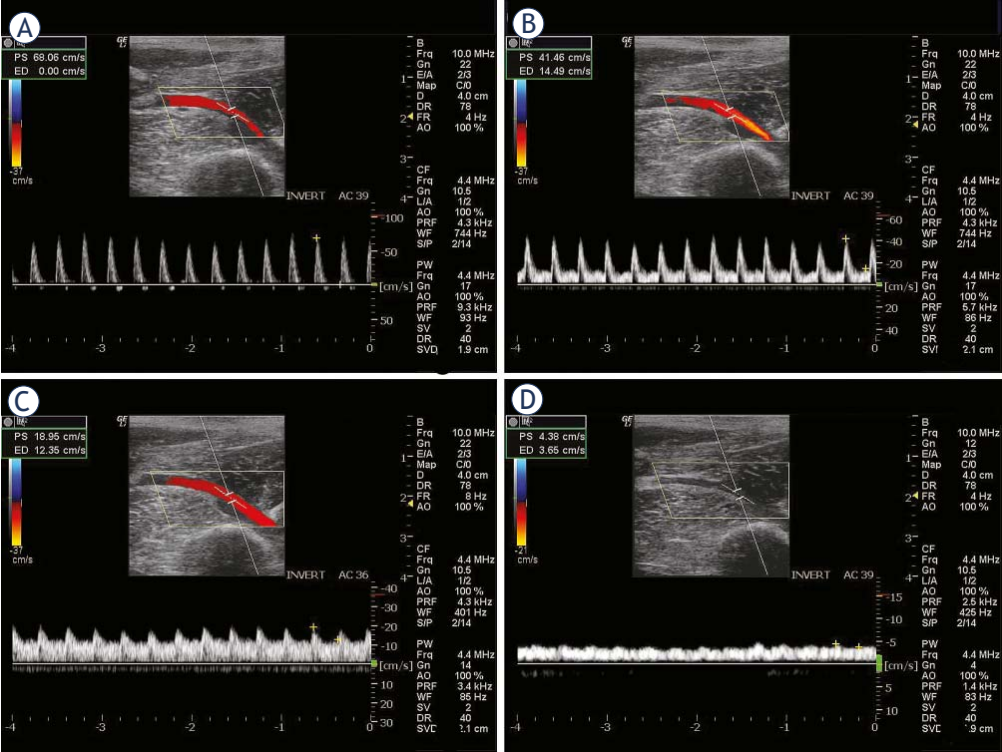
Velocity wave (A–C) of right common femoral artery after experimental abdominal aortic stenosis. A: 1/3 lumen stenosis, B: 1/2 lumen stenosis, C: 2/3 lumen stenosis, D: 3/4 lumen stenosis.

For the velocity of common femoral artery in limb tightening, the systolic principal wave changed slightly and the duration of backward wave was shortened or even disappeared at 1/3 lumen stenosis (Figure 5A). The persistent low amplitude with slight fluctuation, whose direction was coincident with the principal wave, appeared in the diastolic phase at 1/2 lumen stenosis (Figure 5B). The window below the systolic wave vanished at 2/3 lumen stenosis (Figure 5C). The tracing approximately levelled at 3/4 lumen stenosis (Figure 5D).

### Alteration of hemodynamic parameters

The hemodynamic parameters of PSV, MPV, PDV and EDV at the stenosis of different degrees are present in Tables 1,2,3. With the increase of pressure in limb tightening, PSV showed a slightly ascendant tendency, MPV negatively increased after gradually descended to zero, PDV declined to different degrees, and EDV gradually decreased to zero. With the increase of dose in the epinephrine injection, PSV increased slightly at a low dose and then slightly decreased, and MPV negatively increased after gradually decreasing to zero. PDV and EDV gradually decreased to zero. With the increase of stenosis severity in the abdominal aortic wall pinching, there was no statistically significant difference between left and right in changed tendency. PSV was reduced and had linearly negative correlation with stenosis severity (R=0.983, R2=0.967). MPV gradually increased, and its direction reversed when the stenosis severity increased, then diminished when the blood flow was occluded by more than 2/3. However, hemodynamics was slightly changed when the blood flow was occluded by 1/2 or 2/3.

## Discussion

Porcine systolic pressure, diastolic pressure, heart rate and cardiac output, which reflect the characteristics of cardiovascular system, are more similar to those in humans than other experimental animals. Thus, pigs were used in previous studies and the present study.^41^,^42^. When the environmental temperature lowers or the vessels are revealed, the vessels will contract. However, the vessel will dilate if the depth of anaesthesia was altered. The changes in the velocity at the detection site can be detected by ultrasound. In order to achieve the similar velocity to humans, some measures were taken in the operation. A warmer was put besides the animals during the experiment aiming to keep consistent ambient temperature at operation site; the dose of anaesthetic was controlled by a micro-pump after anaesthesia induction aiming to maintain stable effective concentration; the vessels were covered with warm saline gauze once they were exposed aiming to maintain consistent temperature and humidity of the vessels. We all observed repeatedly the velocity tracing in each stage of the experiment for different swine, which removed the runner’s cause of transforming the velocity tracing.

Under normal condition, blood flow produces tension on vessel wall, which varies with the changes in the effective circulating blood volume.^43^ The cardiovascular movement has its own periodicity. On the one hand, the blood flow in the peripheral artery abruptly accelerates with the kinetic energy supplied by the cardiac ejection, and so the velocity wave displays steep ascent in the systolic phase. On the other hand, the cyclic strain of vessel wall increases with a great quantity of blood inflowing the great artery of proximal end rapidly, which promotes the elastic distension of vessel wall because of its elasticity. In the diastolic phase, the heart stops ejecting when the aortic valve closes. The hemokinesis of peripheral artery mainly depends on the inertia at this time. However, the elastic recoil of proximal great artery impulses downstream blood to accelerate, resulting in low amplitude in the velocity wave in the diastolic midanaphase. Peripheral blood loses partial kinetic energy in the process of flow because it does work to overcome the peripheral resistance. The rapid cardiac ejection supplies maximal kinetic energy to the blood in the peripheral artery. Therefore, the velocity waves display systolic principal wave, diastolic secondary wave and wave trough among them.

If the resistance of downstream circulation increases, on one hand, the upstream blood does more work to overcome this resistance simultaneously resulting in lose of more kinetic energy in the process of flow, which further slows the blood flow; on the other hand, the flow of upstream artery is blocked, and its effective circulating blood volume increases accompanied by strengthening of its cyclic strain synchronously, which leads to its elastic expansion. Once the velocity of systolic blood decreases to zero, the vessels elastically contract, producing a reverse blood flow. We identified the diastolic velocity with constant waveform but lowered wave amplitude with the increase of downstream circulation resistance by tightening distal limbs, and the transient reverse velocity wave following the principal wave presented after circulation resistance increased to a certain degree, which resembled the triphasic wave of lower extremity artery in healthy adults.

Adrenaline hydrochloride possesses agonistic effects on α-acceptor and β-receptor. The activated α-acceptor provokes vasoconstriction of skin, mucous membrane and internal organs; the activated β-acceptor excites the skeletal muscle and myocardium, relaxes the tracheal smooth muscle and gastrointestinal smooth muscle, dilates the coronary artery and increases the heart rate. The effect on blood pressure depends on the dose, and the commonly used dose causes the increase of systolic pressure and a slight decline of diastolic pressure, but the high dose significantly increases the blood pressure.^44^ Generally, the dose of agonist leads to increase of systolic and diastolic pressure. On the one hand, it can also result in vasoconstriction of skin, mucous membrane and internal organs, leading to increase of systemic vascular resistance; on the other hand, it enables the contraction of the vessel wall. Our results showed the velocity wave remained constant but the diastolic wave amplitude lowered, and the conspicuous reverse velocity behind the principal wave appeared when the dose increased a certain level.

Both limb tightening and adrenalin hydrochloride can cause the increase of peripheral vascular resistance. Thus, the diastolic wave amplitude lowered but the velocity wave remained constant. The reverse velocity wave appeared after MPV gradually decreased to zero accompanied by the increase of peripheral vascular resistance. These two experiments verified the assumption that peripheral vascular resistance and the elastic recoil of blood vessel had impact on the peripheral artery velocity. Otherwise, the wave amplitude of reverse velocity as a result of a large dose of adrenalin hydrochloride was higher than that after limb tightening, which illustrated the wave amplitude of reverse velocity correlated with vasoconstriction.

When the downstream circulation resistance decreased, the upstream blood did less work to surmount the resistance and subsequently, the drop of kinetic energy in the process of flow was decreased simultaneously, which, in turn, slowed the decrease of blood flow down. Soon after the removal of pressure in limb tightening or discontinuation of adrenalin hydrochloride injection, the reverse velocity wave disappeared because the circulation resistance dropped due to vasodilatation, and the velocity wave restored to the form before experiment. These results demonstrated the diastolic velocity correlated with the downstream circulation resistance, and the reverse velocity appeared because of the elastic recoil of downstream artery.

When the regional effective circulating blood volume diminishes, on one hand, the cyclic strain of vessel wall decreases, as a result of which the elastic recoil of vessel wall weakens; on the other hand, the circulation resistance reduces with the decrease of the vessel pressure. Arterial stenosis is a common cause resulting in the vanishment of distal effective circulating blood volume. The velocity wave of normal lower limb artery displays triphasic. In the experiment, abdominal aortic stenosis of different degrees induced the decrease of effective circulating blood volume of lower extremity, resulting in decrease of the amplitude and the duration of the reverse velocity wave. When the downstream effective circulating blood volume diminishes to a certain degree, the cyclic strain of vessel wall disappears, and then the reverse velocity wave vanishes. If it still diminishes, the circulation resistance decreases because the vessel pressure is reduced, and then the energy loss of hemokinesis decreases, the persistent forward blood emerges in the diastolic phase, presenting nearly flat velocity wave. The characteristics and changes of peripheral artery velocity wave are the results of interactions between systolic function, blood vessel elasticity, effective circulating blood volume and peripheral vascular resistance. The heart rapid ejection produces systolic wave whose altitude is affected by systolic function. The acceleration and deceleration of systolic wave are associated with the circular resistance. The vascular elastic recoil, which pertains to effective circulating blood volume and circular resistance, produces a forward wave in the diastolic midanaphase and a backward wave in the systolic phase. The reverse bloodstream wave in straight artery is a kind of distinctive waveform and hints that the downstream circulating resistance is relatively high. As the result of the interaction between downstream circular resistance and vascular elastic recoil, this wave can be employed to analyze the changes in hemodynamics on the basis of the velocity waves, which may signify some pathological changes of some diseases and their severity, such as stenosis, fistula, and aneurysm.

## Figures and Tables

**TABLE 1.** Hemodynamic parameters of ipsilateral external iliac artery when a hindlimb was tightened in 6 pigs

**Tightening degree**	**PSV (cm/s)**	**MPV (cm/s)**	**PDV (cm/s)**	**EDV (cm/s)**
pre-experiment	36.33±1.77	13.68±1.11	19.31±0.86	13.2±0.45
1	45.87±1.89	6.75±1.24	10.83±1.17	0
2	51.90±3.47	−4.37±0.57	12.27±0.57	0
3	59.72±2.67	−7.48±0.82	8.98±0.45	0
4	51.05±2.52	16.92±1.79	23.17±0.52	15.73±1.03

PSV = peak systolic velocity; MPV = minimum post-principal wave velocity; PDV = peak diastolic velocity; EDV = end diastolic velocity

Note: Group 1, 2, and 3 represent different extents of hindlimb tightening in the experiment on the basis of aesthesis. In addition, no downstream bloodstream was displayed in Group 3, and the constraint was removed in Group 4. For PSV of external iliac artery, there was statistically significant difference except Group 1 and Group 2, Group 2 and Group4 (P<0.05); For MPV and EDV, the difference among these groups was statistically significant (P<0.05); For PDV, there was statistically significant difference except Group 1 and Group 2, Group 1 and Group 3 (P<0.05).

**TABLE 2.** Hemodynamic parameters of external iliac artery after intravenous epinephrine hydrochloride administration in 6 pigs

**Dose**	**PSV (cm/s)**	**MPV (cm/s)**	**PDV (cm/s)**	**EDV (cm/s)**
pre-experiment	36.33±1.77	13.68±1.11	19.31±0.86	13.2±0.45
1/3 ampul	43.97±2.15	10.62±1.38	16.87±0.83	9.57±0.74
1/2 ampul	32.23±1.31	6.12±0.63	13.63±0.65	8.28±0.70
1 ampul	35.43±3.01	−23.53±0.82	0	0
Discontinuation	76.58±8.37	20.68±0.79	24.7±1.49	16.21±2.51

PSV = peak systolic velocity; MPV = minimum post-principal wave velocity; PDV = peak diastolic velocity; EDV = end diastolic velocity

For PSV of external iliac artery, there was statistically significant difference except Group 1/2 ampoule and Group 1 ampoule (P<0.05); For MPV and PDV, the difference among these groups was statistically significant (P<0.05); For EDV, there was statistically significant difference except Group 1/2 ampoule and Group 1/3 ampoule, pre-experiment group and discontinuation group (P<0.05).

**TABLE 3.** Hemodynamic parameters of bilateral common femoral artery in the experiment of abdominal aortic stenosis in 6 pigs

**Degree of stenosis**	**PSV (cm/s)**		**MPV (cm/s)**	
	
	**right**	**left**	**right**	**left**
pre-experiment	90.43±6.31	82.71±6.64	−12.51±0.96	−11.23±0.90
1/3	64.29±3.17	54.42±1.88	−8.51±1.23	−7.35±0.72
1/2	41.57±3.01	36.19±2.84	12.61±1.15	10.88±0.62
2/3	18.06±1.60	14.74±1.60	11.66±1.08	9.67±1.21
3/4	4.24±0.61	4.42±0.25	3.33±0.44	3.49±0.19

PSV = peak systolic velocity; MPV = minimum post-principal wave velocity

For PSV of ipsilateral common femoral artery, there was significant difference among these groups (P<0.05); For MPV, there was statistically significant difference except Group 1/2 stenosis and Group 2/3 stenosis (P<0.05).
